# The effect of athletes' training satisfaction on competitive state anxiety—a chain-mediated effect based on psychological resilience and coping strategies

**DOI:** 10.3389/fpsyg.2024.1409757

**Published:** 2024-09-20

**Authors:** Xiaomei Yu, Yang Yang, Bo He

**Affiliations:** ^1^College of Physical Education, Chengdu Sport University, Eastern New District, Chengdu, China; ^2^College of Physical Education and Health Management, Chongqing University of Education, Nan'an District, Chongqing, China; ^3^College of Physical Education, Chongqing University of Posts and Telecommunications, Nan'an District, Chongqing, China

**Keywords:** training satisfaction, psychological resilience, coping strategies, competitive state anxiety, chain mediation

## Abstract

**Introduction:**

This study aimed to investigate the relationship between athletes' training satisfaction and competitive state anxiety, by examining the mediating roles of psychological resilience and coping strategies. The findings provide a theoretical foundation and practical recommendations for enhancing athletes' training and mental health services.

**Methods:**

A questionnaire was utilized to assess training satisfaction, psychological resilience, coping strategies, and Competitive State Anxiety among a sample of 447 athletes. The data was examined through descriptive statistics, correlation analysis, and structural equation modeling, employing SPSS and the Process 3.5 plug-in.

**Results:**

Training satisfaction had a significant positive effect on psychological resilience (*β* = 0.726, *p*<0.001), while training satisfaction (*β* = 0.178, *p*<0.001) and psychological resilience (*β* = 1.138, *p*<0.001) were found to have a significant positive effect on strategy. Additionally, training satisfaction, psychological resilience, and coping strategies all demonstrated a significant negative effect on competitive state anxiety (*p*<0.001). Training satisfaction indirectly influenced competitive state anxiety through psychological resilience (indirect effect 1), coping strategies (indirect effect 2), and the combined mediating effect of psychological resilience and coping strategies (indirect effect 3), resulting in a total indirect effect of −0.385 [95% CI = (−0.433, −0.337)].

**Discussion:**

In conclusion, enhancing athletes' training satisfaction can help reduce competitive state anxiety by improving psychological resilience and fostering positive coping mechanisms.

## 1 Introduction

In the recently revised Sports Law of the People's Republic of China, Article 24 of Chapter III explicitly emphasizes that the State promotes and backs the advancement of sports training and competitions, enhances athletes' competitive abilities, and implements measures to safeguard athletes' legitimate rights and interests. This legal stipulation underscores the significant value that the state places on enhancing athletes' competitive skills. During training and competition, athletes often experience high levels of competitive state anxiety. Research shows that leading up to major events, ~70% of athletes exhibit symptoms of anxiety, such as insomnia, loss of appetite, and difficulty concentrating (Chen et al., 2023). This anxiety not only impacts athletes' performance, but can also have negative effects on their overall physical and mental wellbeing.

Several studies have demonstrated the significant influence of an athlete's competitive state on their performance during competition (Lee and Shin, 2018; Shin, 2020; Son, 2020). Attaining an athlete's optimal competitive state is essential for reaching a peak level of athletic performance. When an athlete is in this optimal state, both their body and mind can achieve the highest level of readiness, enabling them to perform at their peak during competition. On the contrary, if athletes are in a poor competitive state, their attention, emotional control, and motor skill execution levels will be affected to varying degrees, resulting in a decline in performance (Hanton et al., 2004). Moreover, prolonged high levels of competitive state anxiety may also contribute to mental health issues in athletes, such as depression and anxiety disorders (Gucciardi and Gordon, 2009). Athletes with high levels of competitive state anxiety may experience this due to various factors, including intense competition pressure, low self-efficacy, and excessive fixation on outcomes (Li and Cao, 2016). Notably, athletes' contentment with the training process could significantly impact this. When athletes are satisfied with the training process, they are more likely to develop strong self-confidence and self-efficacy, which can help reduce anxiety levels during competition (Zhang, 2014). Furthermore, the psychological resilience and coping strategies of athletes may also serve as crucial mediators between training satisfaction and competitive state anxiety. Athletes with high levels of psychological resilience are more adept at handling the challenges and pressures of training and competition, leading to reduced anxiety levels (Gucciardi and Gordon, 2009). Positive coping strategies, such as problem-solving and seeking social support, can also assist athletes in alleviating competitive state anxiety (Nicholls et al., 2008).

### 1.1 The effect of training satisfaction on competitive state anxiety

The impact of athlete training satisfaction on competitive state anxiety has been substantiated by multiple studies. Chelladurai and Riemer (1997) introduced a model of athlete satisfaction, asserting that an athlete's contentment with the training regimen plays a crucial role in shaping their psychological state and competitive outcomes. It should be clarified that athlete satisfaction and training satisfaction are two related but not identical concepts. Athlete satisfaction is a broader concept that includes satisfaction with one's overall performance, while training satisfaction specifically refers to satisfaction with the training environment and process. This study focuses on training satisfaction. Athletes' training satisfaction encompasses various dimensions. Prabowo (2024) identified four key aspects: training quality, mental and emotional wellbeing, competitive performance, and competitive experience. On the other hand, Chinese scholar Ronghai et al. (2013) categorized it into four groups: training conditions, coaching elements, individual circumstances, and logistical factors. This study focuses on the latter model. Carpentier and Mageau (2016) research revealed that athletes' training satisfaction is significantly influenced by their sports experiences during training. Regarding coaching factors, Baker et al. (2000) and Cho et al. (2019) emphasized the positive correlation between the quality of the coach-athlete relationship and athletes' training satisfaction, highlighting how a strong relationship can enhance satisfaction. Studies by Balaguer et al. (1999) and Reinboth and Duda (2006) demonstrated a link between athletes' goal orientation and their training satisfaction, showing that task-oriented athletes tend to exhibit higher levels of satisfaction. Lastly, MacIntosh et al. (2020) found a notable connection between the event service environment and athletes' training satisfaction. Furthermore, relevant research indicates that athletes who are content with the training environment, coaching guidance, training regimen, and intensity are more inclined to uphold a positive mental state and excel in their performance during the game. Conversely, athletes who are discontent with the training process may encounter negative emotional responses, such as anxiety and anger, which can impact their competitive performance (Smith et al., 1995; Liwei et al., 2003; Xie, 2008; Campo et al., 2012). Reverdito et al. (2023) highlighted that athlete satisfaction with their experience can lead to increased motivation for consistent training, resulting in improved performance. Similarly, Khorram (2023) emphasized that athlete satisfaction with training and coaching can enhance their willingness to learn and develop new skills, ultimately enhancing their quality and abilities as athletes.

These findings can be interpreted through the lens of self-determination theory (Deci and Ryan, 2000). According to this theory, individuals are more likely to experience positive emotions and demonstrate increased intrinsic motivation and psychological wellbeing when their fundamental psychological needs, such as autonomy, competence, and relatedness, are fulfilled. In athletic training scenarios, when athletes are content with the training process, their autonomy, competence, and sense of relatedness needs are more likely to be fulfilled. This, in turn, enhances positive emotional experiences and lowers levels of athletic state anxiety. Furthermore, training satisfaction may impact athletes' competitive state anxiety by influencing their stress evaluation mechanisms. According to Lazarus and Smith (1988) cognitive-emotional-relational theory, an individual's assessment of a stressor influences their emotional reactions and coping behaviors. Athletes who are content with the training process tend to view competition as a challenge rather than a threat, leading to lower levels of competitive state anxiety (Nicholls et al., 2012). In summary, athlete training satisfaction may significantly influence competitive state anxiety through various mechanisms, such as meeting basic psychological needs, promoting positive emotional experiences, and impacting stress evaluation processes. Therefore, the present study proposes:

Hypothesis 1: Athlete training satisfaction has a negative and significant effect on competitive state anxiety.

### 1.2 The mediating role of psychological resilience

Resilience is the capacity to encounter adversities, setbacks, and challenges, and not only recover to one's previous state but also achieve greater success, wellbeing, and mental fortitude (Fletcher and Sarkar, 2013; Everly et al., 2015). Psychological resilience and mental toughness are closely related concepts, and in certain contexts, these terms can be used interchangeably (Arora et al., 2022; Wu et al., 2023). For the sake of clarity and consistency, this study employs a unified term: psychological resilience. Research has demonstrated that psychological resilience varies significantly across different sports (Gucciardi et al., 2011), prompting researchers to develop sport-specific psychological resilience scales. These scales have been tailored for various sports, including cricket (Gucciardi and Gordon, 2009; Sharma and Sharma, 2015), Kuçuk Kiliç (2020), and competitive tennis (Cowden et al., 2016a). Athletes with high levels of psychological resilience are better equipped to handle the demands of training and competition, maintain positive emotional states and motivation levels, and consequently decrease competitive state anxiety (Gucciardi et al., 2015). Enhancing personal skills/assets like coping, problem-solving, self-efficacy, emotional control, and intelligence can significantly boost athletes' psychological resilience. Furthermore, research has indicated that social support can enhance resilience (Wang et al., 2015). Studies have demonstrated a strong connection between athletes' training satisfaction and their psychological resilience. Lu et al. (2016) and Anthony et al. (2018) conducted research with college athletes, revealing that satisfaction with the training environment, coaching support, and teammate relationships were significant predictors of psychological resilience levels. Similarly, Butt et al. (2010) also found that coaching played a critical role in the development of psychological resilience. This included coaching support, coaching attributes, and coaching practices. Furthermore, Beattie et al. (2019) emphasized the importance of self-regulated training behaviors in the physical and mental development of athletes, with training behaviors acting as a mediator between self-reported psychological resilience and psychological resilience behaviors in swimming. These studies indicate that when athletes are content with their training, they are more likely to cultivate and sustain high levels of psychological resilience. psychological resilience, in return, can assist athletes in effectively managing stress during both training sessions and competitions, as well as in decreasing levels of competitive state anxiety. In a separate study by Hosseini and Besharat (2010) on elite athletes, it was discovered that psychological resilience had a significant negative correlation with athletic state anxiety, indicating that higher levels of psychological resilience were associated with lower levels of athletic state anxiety. Similarly, Hannibalsson (2018) study revealed that psychological resilience significantly predicted athletes' pre-competition state anxiety. Athletes with high levels of psychological resilience displayed lower cognitive and physical state anxiety prior to competition. These results indicate that psychological resilience could potentially act as a mediator between athletes' training satisfaction and competitive state anxiety. This mediating effect can be derived from the cognitive-emotion regulation theory (Garnefski et al., 2001). According to this theory, an individual's cognitive appraisal and emotion regulation strategies play a mediating role between stressors and emotional responses. In sports contexts, psychological resilience can be seen as a beneficial cognitive evaluation and emotion regulation technique that assists athletes in perceiving challenges in training and competition as manageable and in adopting positive coping mechanisms, thus reducing levels of competitive state anxiety (Fletcher and Sarkar, 2012). In essence, psychological resilience could serve as a significant mediating factor between athletes' training satisfaction and competitive state anxiety. Therefore, the present study proposes:

Hypothesis 2: psychological resilience has a significant mediating effect between athletes' training satisfaction and competitive state anxiety, i.e., training satisfaction reduces competitive state anxiety by increasing the level of psychological resilience.

### 1.3 The mediating role of coping strategies

Coping strategies, defined as cognitive and behavioral efforts employed by individuals when dealing with stressful situations (Lazarus and Smith, 1988), could potentially serve as a significant mediator between athletes' satisfaction with their training and their levels of competitive state anxiety. According to the cognitive-emotional-relational theory proposed by Lazarus and Smith (1988), an individual's cognitive evaluation of a stressor and the coping mechanisms they utilize are crucial factors that impact their emotional reactions. In athletic contexts, an athlete's satisfaction with the training process may impact their selection and implementation of coping strategies, subsequently influencing the level of competitive state anxiety (Nicholls et al., 2012). Research has demonstrated a close relationship between athletes' training satisfaction and their utilization of coping strategies. Chen and Wei (2014) research revealed that coping styles play a mediating role in the connection between athletes' self-efficacy and cognitive trait anxiety. Furthermore, it was observed that athletes' levels of trait anxiety can be effectively regulated by enhancing their self-efficacy and acquiring suitable coping mechanisms. Similarly, Kristjánsdóttir et al. (2018) also found in their study that adolescent elite athletes' satisfaction with the training process was significantly and positively correlated with their use of positive coping strategies, such as working hard and seeking social support. Gaudreau and Blondin (2002) conducted a study on college athletes and discovered that the utilization of positive coping strategies, such as positive cognitive reappraisal and problem-solving, showed a significant negative correlation with pre-competition state anxiety. In contrast, the use of negative coping strategies, like avoidance and self-blame, exhibited a significant positive correlation with pre-competition state anxiety. Kato (2015) and Kraaij and Garnefski (2019) found that distraction seeking, positive approaching, and social support seeking were negatively correlated with symptoms of depression and anxiety, while withdrawal and neglect were positively correlated with these symptoms. These findings suggest that coping strategies may mediate the relationship between athletes' training satisfaction and competitive state anxiety. This mediating effect can be explained by cognitive-emotion regulation theory Garnefski et al. (2001), which posits that cognitive-emotion regulation strategies adopted by individuals play a role in mediating between stressors and emotional responses. In sports contexts, positive coping strategies such as positive cognitive reappraisal and problem-solving can assist athletes in evaluating stressors during training and competition as manageable, enabling them to take constructive steps to deal with these stressors. This in turn helps in reducing levels of competitive state anxiety (Nicholls et al., 2012). In essence, coping strategies could potentially serve as a crucial mediator in the relationship between athletes' satisfaction with their training and their experience of competitive state anxiety. Therefore, the present study proposes:

Hypothesis 3: Coping strategies had a significant mediating effect between athletes' training satisfaction and competitive state anxiety, i.e., training satisfaction affected the level of competitive state anxiety by influencing the selection and use of coping strategies.

### 1.4 Chain mediation of psychological resilience and coping strategies

Before delving into the chain mediation effect of psychological resilience and coping strategies, it is essential to first differentiate between the two concepts. Psychological resilience is defined as the dynamic process and outcome of an individual's ability to effectively adapt when confronted with adversity, stress, or trauma (Luthar et al., 2000). It is viewed as a relatively stable personality trait that reflects an individual's overall adaptability and psychological strength in the face of challenges (Fletcher and Sarkar, 2013). On the other hand, coping strategies refer to the specific cognitive and behavioral efforts that individuals employ in response to particular stressful situations (Lazarus and Folkman, 1984). Despite their distinctiveness, research indicates that individuals with high psychological resilience are more likely to utilize active coping strategies (Secades et al., 2016). In addition to the mediating effects of psychological resilience and coping strategies between athletes' training satisfaction and athletic state anxiety, this study further hypothesized that psychological resilience and coping strategies may have a sequential mediating effect between the two. This hypothesis is supported by both theoretical and empirical evidence. psychological resilience, as a personal trait, can impact an individual's cognitive appraisal and selection of coping mechanisms during stressful situations (Fletcher and Sarkar, 2013; Crust and Azadi, 2010). This hypothesis is supported by both theoretical and empirical evidence. psychological resilience, as a personal trait, can impact an individual's cognitive appraisal and selection of coping mechanisms during stressful situations (Fletcher and Sarkar, 2013; Crust and Azadi, 2010). Previous research has shown a significant correlation between psychological resilience and coping strategies. For instance, Secades et al. (2016) found that psychological resilience was positively associated with task-oriented coping strategies (e.g., hard work, positive thinking) and negatively associated with emotion-oriented coping strategies (e.g., emotional catharsis, avoidance). Similarly, Cowden et al. (2016b) found in their study of competitive tennis players that psychological resilience was significantly and positively associated with positive coping strategies, such as positive cognitive reappraisal and problem-solving. The research also indicated that when athletes are content with their training process, their psychological resilience tends to rise, leading to higher levels of optimism, confidence, and focus (Sheard and Golby, 2006). Higher levels of psychological resilience may assist athletes in perceiving stressful situations during training and competition as challenges rather than threats, enabling them to utilize positive coping strategies (Fletcher and Sarkar, 2012). The implementation of these positive coping strategies can ultimately aid athletes in effectively managing their emotional state and decreasing levels of competitive state anxiety (Nicholls et al., 2016). This chain-mediated effect can be understood through the lens of cognitive-emotion regulation theory Garnefski et al. (2001) and psychological resilience theory Fletcher and Sarkar (2012) from an integrated perspective. Cognitive-emotion regulation strategies, as proposed by the cognitive-emotion regulation theory, serve as mediators between stressors and emotional responses in individuals. According to the psychological resilience theory, psychological resilience can impact an individual's assessment of stressors and their choice of coping strategies. By combining these two theories, psychological resilience and coping strategies can be seen as crucial factors that mediate the relationship between stressors (such as training satisfaction) and emotional responses (like competitive state anxiety). This study proposes a hypothesis that psychological resilience and coping strategies have a chain-mediated effect on athletes' training satisfaction and competitive state anxiety, building upon theoretical and empirical foundations.

Hypothesis 4: psychological resilience and coping strategies have a chain-mediated effect between athletes' training satisfaction and competitive state anxiety, i.e., training satisfaction reduces competitive state anxiety levels by sequentially increasing levels of psychological resilience and promoting the use of positive coping strategies.

## 2 Research methodology

### 2.1 Participants

A total of 447 samples were collected for this study, with 241 (53.9%) males and 206 (46.1%) females. The sample size for the age group of 12–15 years old was 52 (11.6%), while the age group of 15–18 years old had the largest proportion with 268 samples (60%). Additionally, there were 61 samples (13.6%) in the age group of 18–22 years old, and 66 samples (14.8%) for those aged 22 years old and above. The study found that the majority of research participants were involved in athletics and ball sports, with 137 people (30.6%) and 174 people (38.9%) respectively. Additionally, 94 participants were at an amateur sports level (No official grade certification) (21%), 216 were at level 3 (Elementary level, county competition winners) (48.3%), and 74 were at level 2 (Intermediate level, municipal competition winners) (16.6%). A small portion of individuals hold sport grades of Grade 1 (Senior level, provincial competition awards), Fitness (Professional level, national competition winners), and International (International competition awards). The majority of athletes have undergone training for a period of 3–5 years, with 217 individuals (48.5%), followed by 1–3 years, with 153 individuals (34.2%).

### 2.2 Research tools

#### 2.2.1 Collegiate athletes training satisfaction scale (CA-TSS)

Developed by Ronghai et al. (2013), the Mixed Chinese and English scale is used to evaluate athletes' satisfaction with the training environment, content, and outcomes. It includes various items that assess athletes' perceptions of training quality and their satisfaction levels, Each entry was rated on a Likert scale (1 = “very dissatisfied” to 5 = “very satisfied”). The total score of the scale reflects the athlete's overall training satisfaction and provides a quantitative basis for subsequent research.

#### 2.2.2 Sports competition anxiety test scale (SCAT)

Developed in 1990 by Martens (1990), the Sport Competition Anxiety Test (SCAT) is designed to assess an athlete's level of competitive trait anxiety during performance situations. The questionnaire consists of 15 statements that athletes use to rate the frequency of their feelings in competitive settings. Ten of the statements specifically measure anxiety-related symptoms, while the remaining five statements are included to mitigate internal response set bias. The scale is scored by converting responses into numerical scores (1 = “rarely”; 2 = “sometimes”; 3 = “usually”) to calculate a total anxiety score. Scores over 24 indicate that an athlete may have high levels of competitive trait anxiety.

#### 2.2.3 Simple coping style scale (SCSQ)

This scale consists of 20 items and utilizes a four-level scoring system (0 = “do not take it,” 1 = “take it occasionally,” 2 = “take it sometimes,” 3 = “take it often”). It is divided into active coping and negative coping dimensions. The positive coping dimension represents the constructive strategies used by the individual in response to stress, whereas the negative coping dimension signifies the maladaptive strategies. An individual's coping tendency is determined by the difference between the positive coping criterion score and the negative coping criterion score, which is then transformed using a *Z*-score. This score indicates the coping style that an individual is inclined to adopt during times of stress (Yanin, 1998).

#### 2.2.4 Brief psychological resilience scale (CD-RISC-10)

Developed by Campbell-Sills et al. (2009) and Stein, the simplified version of the original 25-item CD-RISC scale consists of 10 entries. Respondents' total scores range from 0 to 40, reflecting their level of psychological resilience. The scale score is determined by the respondent's self-assessment of each item, with “never” equaling 0 points, “rarely” equaling 1 point, “sometimes” equaling 2 points, “often” equaling 3 points, and “almost always” equaling 4 points. A higher total score indicates greater psychological resilience in the individual.

### 2.3 Research procedures

This study employed a series of rigorous statistical methods and procedures to ensure the reliability and validity of the results. Initially, SPSS 26.0 was utilized for descriptive statistics and Pearson correlation analysis to investigate the fundamental characteristics and relationships between variables. To address potential multicollinearity issues, the study employed the variance inflation factor (VIF) method for covariance testing and eliminated variables with significant covariance problems (VIF>10), thereby strengthening the model's robustness. For mediation effect analysis, Model 6 in the process plug-in developed by Hayes (2017) was utilized to assess the chain mediation effect. To enhance the accuracy of the significance test for the mediation effect, the study implemented the bias-corrected percentile Bootstrap method, using the 99% confidence interval as a benchmark; if the confidence interval excludes the value 0, it signifies statistical significance (Erceg-Hurn and Mirosevich, 2008).

## 3 Findings

### 3.1 Common methodology bias

To investigate the impact of common method bias on the study findings, the current research employed the Harman one-way test. All measures were included in an exploratory factor analysis to determine if a single factor accounted for the majority of variance without rotation. The analysis revealed nine distinct factors, with the first factor explaining 34.408% of the variance, falling below the critical threshold of 40%. The findings indicate that the measurement items, despite being based on self-reports from subjects, were not significantly influenced by common method bias. Therefore, the study results accurately represent the genuine relationship between the variables, rather than being distorted by measurement methods. This discovery strengthens the reliability and validity of the results, providing a robust basis for further discussion. Of course, future studies should continue to explore various methods for controlling common biases, such as integrating objective measurement indicators and implementing longitudinal designs, in order to enhance the study's rigor and persuasiveness.

### 3.2 Descriptive statistics and variable correlation analysis

The study performed descriptive statistics and correlation analysis on the variables using SPSS 26.0. Table 1 displays the specific mean, standard deviation, and correlation coefficient between the variables. The data indicated significant positive correlations among all variables, with the exception of a significant negative correlation between training satisfaction, psychological resilience, coping strategies, and athletes' competitive state anxiety.

**Table 1:** Descriptive statistics and correlation matrix for each variable.

	**Mean**	**Standard deviation**	**Training satisfaction**	**Psychological resilience**	**Coping strategies**	**Competitive state anxiety**
Training satisfaction	3.015	0.799	1			
Psychological resilience	1.850	0.928	0.726^***^	1		
Coping strategies	1.723	0.401	0.660^***^	0.833^***^	1	
Competitive state anxiety	2.068	0.417	-0.776^***^	-0.804^***^	-0.732^***^	1

^***^p < 0.001.

Training satisfaction: CA-TSS scale, Athletic State Anxiety: SCAT scale, Coping Strategies: SCSQ scale, Psychological Resilience: CD-RISC-10 scale.

### 3.3 Chain mediation effect test

**Figure 1:**
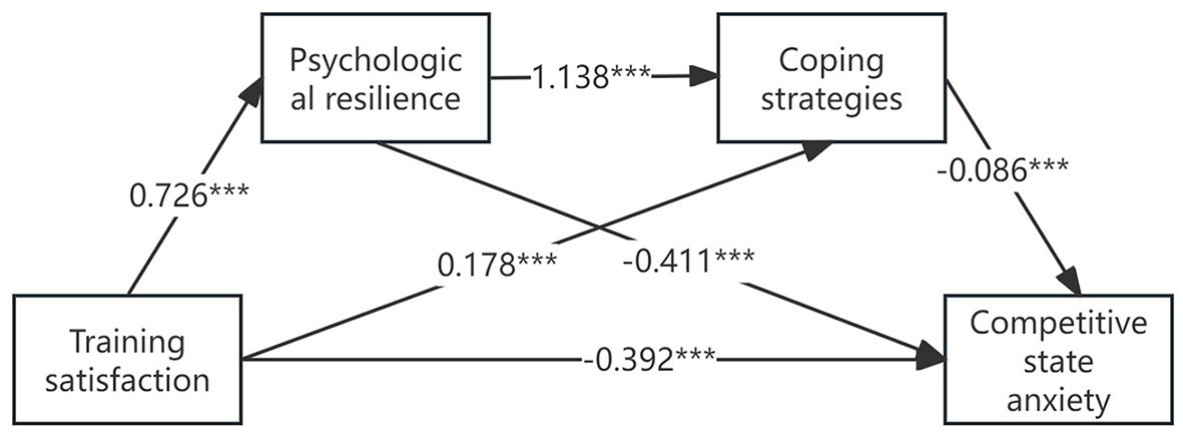
Concept of the moderating effect hypothesis. ***p < 0.001.

To examine the relationship between training satisfaction, competitive state anxiety, psychological resilience, and coping strategies, this study utilized SPSS 26.0 and PROCESS 3.5 plug-ins to perform stepwise regression analysis. The study first assessed the influence of training satisfaction on psychological resilience, then analyzed the combined effects of training satisfaction and psychological resilience on coping strategies, and finally explored the collective impact of training satisfaction, psychological resilience, and coping strategies on competitive state anxiety. The regression analysis results indicate that training satisfaction has a significant positive impact on psychological resilience (β = 0.726, *t* = 22.269, *p* < 0.001), with the model explaining 52.7% of the variance (*R*^2^ = 0.527, *F* = 495.889). Further analysis reveals that both training satisfaction (β = 0.178, *t* = 3.103, *p* < 0.001) and psychological resilience (β = 1.138, *t* = 19.807, *p* < 0.001) significantly influence coping strategies, explaining 70.1% of the variance in the model (*R*^2^ = 0.701, *F* = 519.271). Additionally, the study finds that training satisfaction (β = -0.392, *t* = -10.804, *p* < 0.001), psychological resilience (β = -0.411, *t* = -8.345, *p* < 0.001), and coping strategies (β = -0.086, *t* = -2.907, *p* < 0.001) all have a significant negative impact on competitive state anxiety, explaining 73.0% of the variance in the model (*R*^2^ = 0.730, *F* = 400.135) (Figure 1). These results suggest that higher training satisfaction leads to stronger psychological resilience, more effective coping strategies, and lower levels of competitive state anxiety among athletes (see Table 2).

**Table 2:** Regression equations for chained intermediaries.

**Regression equation (*****N*** **= 447)**		**Fitting index**			**Coefficient and significance**	
**Outcome variables**	**Predictor variables**	**R**	**R** ^^2^^	**F**	β	**t**
Psychological resilience	Constants	0.726	0.527	495.889	0.000	0.000
	Training satisfaction				0.726^***^	22.269
Coping Strategies	Constants	0.837	0.701	519.271	0.078^**^	1.974
	Training satisfaction				0.178^***^	3.103
	psychological resilience				1.138^***^	19.807
Competitive State Anxiety	Constants	0.855	0.730	400.135	0.007	0.271
	Training satisfaction				-0.392^***^	-10.804
	Psychological resilience				-0.411^***^	-8.345
	Coping Strategies				-0.086^***^	-2.907

^***^p < 0.001, ^**^p < 0.05.

Mediation effect analysis using the Bootstrap methodology showed a significant total indirect effect of training satisfaction on competitive state anxiety of -0.385 [Boot SE=0.025, 95% CI = [-0.433, -0.337]], with a relative mediation effect of 50.07%. The study found that the indirect effect of training satisfaction on competitive state anxiety through psychological resilience was -0.298 [Boot SE = 0.035, 95% CI = [-0.367, -0.227]], accounting for 38.75% of the mediation effect. Additionally, the indirect effect of training satisfaction on competitive state anxiety through coping strategies was -0.015 [Boot SE = 0.008, 95% CI = [-0.032, -0.002]], representing a relative mediation effect of 1.95%, and the chain mediated effect of training satisfaction on competitive state anxiety via psychological resilience and coping strategies (indirect effect 3) was -0.071 [Boot SE = 0.029, 95% CI = [-0.130, -0.018]], with a relative mediation effect of 9.23%. The relative mediation effect was 9.23% (see Table 3).

**Table 3:** Bootstrap analysis of the mediation effects test.

	**Effect**	**Boot SE**	**Boot LL CI**	**Boot UL CI**	**Relative mediation effect**
Total indirect effect	−0.385	0.025	−0.433	−0.337	50.07%
Indirect effect 1	−0.298	0.035	−0.367	−0.227	38.75%
Indirect effect 2	−0.015	0.008	−0.032	−0.002	1.95%
Indirect effect 3	−0.071	0.029	−0.130	−0.018	9.23%

Indirect effect 1: Training satisfaction → psychological resilience → Competitive State Anxiety.

Indirect effect 2: Training satisfaction → Coping Strategies → Competitive State Anxiety.

Indirect effect 3: Training satisfaction → psychological resilience → Coping Strategies → Competitive State Anxiety.

To further investigate the causal relationship between psychological resilience and coping strategies, and to address potential limitations of cross-sectional data, this study conducted an alternative model by reversing the order of the two mediating variables. In this alternative model, coping strategies were considered as the first mediating variable (M1) and psychological resilience as the second mediating variable (M2). The results of the analysis revealed that the total indirect effect amounted to 49.61% [-0.385, 95% CI = [-0.431, -0.336]], which was largely in line with the original model. However, there were significant changes in the contribution of each indirect effect path: the value of indirect effect 1 (training satisfaction ← coping strategies ← competitive state anxiety) was -0.087 [95% CI = [-0.152, -0.024]], representing a mediation effect of 11.21%; the value of indirect effect 2 (training satisfaction ← psychological resilience ← competitive state anxiety) was -0.128 (95% CI = [-0.173, -0.087]); and the value of indirect effect 3 (training satisfaction ← coping strategies ← psychological resilience ← competitive state anxiety) was -0.170 [95% CI = [-0.216, -0.126]], with a relative mediation effect of 21.91%.

The comparative analysis of these two models reveals an interesting phenomenon: regardless of the order in which psychological resilience and coping strategies are arranged, both play a crucial mediating role in the impact of training satisfaction on competitive state anxiety. However, when psychological resilience is considered as the initial mediating variable, its independent mediating effect is more pronounced (38.75 vs. 16.49%); conversely, when coping strategies are the first mediating variable, the chain mediating effect becomes more prominent (21.91 vs. 9.23%). This discovery not only underscores the significance of psychological resilience and coping strategies in competitive sports psychology but also offers fresh insights into their intricate interplay. While the cross-sectional nature of this study limits the ability to establish causality definitively, this comparative analysis approach presents valuable avenues for future longitudinal research and enriches our understanding of the dynamic relationships between these psychological variables.

## 4 Discussion

This study examines the effects of athletes' training satisfaction on competitive state anxiety and investigates the mediating roles of psychological resilience and coping strategies. A questionnaire survey method was used to conduct large-scale research athletes. Utilizing structural equation modeling, the study revealed that training satisfaction exhibited a significant negative predictive impact on athletic state anxiety. Additionally, psychological resilience and coping strategies were identified as chain mediators between training satisfaction and athletic state anxiety. More specifically, training satisfaction was found to positively influence psychological resilience, which in turn positively influenced the use of positive coping strategies. Ultimately, positive coping strategies were associated with a negative prediction of athletic state anxiety. This series of results elucidated the internal mechanism by which training satisfaction influences Competitive State Anxiety.

### 4.1 Training satisfaction reduces competitive state anxiety

This study revealed that athletes' training satisfaction has a significant negative effect on competitive state anxiety, and hypothesis 1 was established. This finding aligns with previous research and adds empirical evidence to the association between athletes' training satisfaction and competitive state anxiety. Training satisfaction reflects athletes' subjective evaluation of various aspects of the training process, including training content, intensity, atmosphere, and interaction with coaches (Chelladurai and Riemer, 1997). When athletes are satisfied with their training, they are more likely to be satisfied with the overall training process. When athletes are satisfied with their training, they tend to exhibit increased levels of intrinsic motivation, self-efficacy, and positive emotions (Treasure et al., 2007). Conversely, athletes experiencing dissatisfaction with their training may feel frustrated, disappointed, and doubtful of their abilities, leading to an increase in competitive state anxiety (Woodman and Hardy, 2003). According to the cognitive appraisal theory proposed by Lazarus and Smith (1988), individuals' perceptions of stressful events and how they appraise them can significantly influence their emotional response. When athletes perceive their training positively, they tend to see challenges as opportunities for growth rather than threats. This positive mindset can effectively decrease competitive state anxiety among athletes. Conversely, athletes who are dissatisfied with their training are more inclined to view training stress as a burdensome challenge, leading to an exacerbation of anxiety levels (Xie, 2008). Previous studies have demonstrated the significance of training satisfaction in influencing athletes' anxiety levels. Additionally, research has shown a correlation between training satisfaction and competitive state anxiety. Ryska and Yin (1999) investigated the impact of athletes' perceptions of coaching support on pre-competition anxiety among high school athletes, revealing that greater coaching support was associated with lower levels of pre-competition anxiety. Baker et al. (2000) also found that a negative relationship between the coach and athlete can lead to an increase in the athlete's anxiety levels. Furthermore, Ramis et al. (2017) discovered that controlling coaching behaviors had a significant correlation with athletes' competitive trait anxiety. On the other hand, autonomy-supportive coaching behaviors did not show a significant association with trait anxiety. The study also revealed that coaches' controlling styles, influenced by controlled motivation, positively predicted athletes' perceived competitive anxiety. Cho et al. (2019) also recommended that coaches should offer less controlling instructions to help decrease athletes' anxiety and burnout.

### 4.2 Psychological resilience partially mediates the relationship between training satisfaction and competitive state anxiety

In this study, psychological toughness was found to mediate the relationship between training satisfaction and competitive state anxiety, and hypothesis 2 was established. This finding aligns with prior research in the sports domain, indicating that psychological resilience is strongly associated with stress management, emotion regulation, and athletic performance in athletes (Gucciardi et al., 2015). Hosseini and Besharat (2010) discovered a negative correlation between psychological resilience and athletes' competition stress, as well as a positive correlation with sports performance. These findings indicate that psychological resilience could impact athletes' emotional wellbeing and athletic achievements through its influence on stress evaluation and management. In a study conducted by Hu et al. (2014), qualitative research on elite athletes revealed that the factors influencing psychological resilience can be categorized into team culture, social support, athletic ability, coping strategies, and stress-resistant personality traits. Further analysis showed that athletes' satisfaction with their training impacts their psychological resilience, subsequently influencing their competitive state anxiety. This mediating process is grounded in cognitive appraisal theory (Lazarus and Smith, 1988). According to this theory, individuals engage in primary appraisal (assessing the nature and significance of the event) and secondary appraisal (assessing their coping resources and abilities) when faced with a stressful event. When athletes are satisfied with their training, they may be more likely to view difficulties and challenges in training as positive and controllable, thereby boosting their self-efficacy and coping confidence. This positive cognitive appraisal can enhance athletes' psychological resilience, leading to stronger adaptive ability and emotional stability when facing training stress, ultimately reducing competitive state anxiety. Rong et al. (2020) further supported this notion, revealing that coaching support behavior significantly impacts athletes' psychological resilience and is a crucial factor in determining athletes' training satisfaction.

### 4.3 Coping strategies partially mediate the relationship between training satisfaction and competitive state anxiety

In this study, it was found that coping strategies mediated the relationship between athletes' training satisfaction and competitive state anxiety, thus confirming Hypothesis 3. This outcome aligns with prior research indicating that utilizing positive coping strategies can mitigate the adverse impact of stressful situations on an individual's psychological wellbeing (Yaacob et al., 2019; Thorne et al., 2013) discovered a significant relationship between coping strategies (positivity, distraction, avoidance, and support-seeking) and anxiety symptoms. Furthermore, Doron et al. (2013) discovered through cluster analysis that adaptive copers exhibited higher levels of self-esteem and lower levels of trait anxiety in comparison to low-adaptive and avoidant copers. Additionally, their further analysis indicated that athletes' satisfaction with their training impacts competitive state anxiety by influencing their selection of coping strategies. Athletes who were satisfied with the training process tended to utilize positive coping strategies like positive reappraisal and problem-solving, while being less inclined to resort to negative strategies such as avoidance and self-blame (Nicholls et al., 2016). Positive coping strategies aid athletes in managing their emotions and maintaining a positive psychological state, ultimately reducing competitive anxiety (Dias et al., 2012). This process can be characterized as a cognitive-emotional-behavioral process. Athletes who are satisfied with their training are more likely to have positive cognitive appraisals, experience positive emotions, and adopt positive and adaptive behavioral strategies. This ultimately leads to a reduction in competitive state anxiety. It is important to note that while coping strategies played a mediating role in the connection between training satisfaction and competitive anxiety, their relative mediating effect was lower compared to that of psychological resilience. This indicates that psychological resilience could be a more fundamental and consistent internal resource than coping strategies. As suggested by Fletcher and Sarkar (2012), psychological resilience is a positive and enduring personality trait, while coping strategies are more influenced by specific situations. Therefore, psychological resilience may play a more fundamental moderating role in the coping process. This study's findings have significant implications for understanding athletes' mental health. The results suggest that in addition to targeting specific coping strategies, it is crucial to prioritize the cultivation and strengthening of psychological resilience in athletes.

### 4.4 Chain mediating effects of psychological resilience and coping strategies between training satisfaction and competitive state anxiety

An important finding of this study is that, there is a significant correlation between psychological resilience and coping strategies, psychological resilience positively affects coping strategies, and that psychological resilience and coping strategies play a chain mediating role between athletes' training satisfaction and athletic state anxiety, Hypothesis 4 is valid. This result reveals the intrinsic link between psychological resilience and coping strategies and their synergistic effects in regulating athletes' psychological states. psychological resilience, as a positive personality trait, has a significant impact on an individual's coping style. Individuals with high levels of psychological resilience tend to adopt more positive and adaptive coping strategies (Connor and Davidson, 2003). Research in the field of sports has also shown a positive association between psychological resilience and positive coping strategies, such as problem-solving and cognitive reappraisal, while a negative association with negative coping strategies, like avoidance and self-blame, has been observed. These studies suggest that psychological resilience may influence individuals' psychological responses to stress by influencing the choice of coping strategies. Specifically, Kaiseler et al. (2009) found that overall psychological resilience and its components were positively associated with problem-focused coping strategies, but negatively associated with emotion-focused and avoidance coping strategies. Athletes with high levels of psychological resilience were found to manage stress more effectively (Jones et al., 2007; Kaiseler et al., 2009). The effectiveness of their coping is dependent on the type of coping strategy they utilize. Those with high psychological resilience reported more positive outcomes when employing problem-focused coping strategies, while they exhibited less effective coping when resorting to emotion-focused or avoidance strategies. In addition, Nicholls et al. (2008) also demonstrated that athletes with higher levels of psychological resilience tend to use problem-solving based or proximity-based coping strategies more frequently, such as mental imagery, effort exertion, thought control, and logical analysis, while using avoidance-based coping strategies less frequently, including distancing, distraction, and resignation. Further analysis revealed that athletes' training satisfaction initially influenced their level of psychological resilience, which subsequently impacted the use of coping strategies and ultimately competitive anxiety.

## 5 Limitations and prospects

Although the current study yielded meaningful findings, there are still limitations that should be addressed in future research. This is reflected in: (1) the cross-sectional design of this study does not allow for the identification of causal relationships between variables; (2) the subjects were mainly Chinese athletes, so the generalizability of the findings needs to be further strengthened; (3) the study relied mainly on the self-report questionnaire measurements, so there may be subjective bias.

This study contributes to the existing research on the influence of athletes' training satisfaction on competitive state anxiety, offering new insights for enhancing mental health services for athletes. Future research could delve deeper into the impact of psychological resilience and coping strategies, including cognitive assessment and metacognition, by refining research methodologies. Simultaneously, the research perspective can be broadened from the individual level to the interpersonal and organizational levels to investigate the impact of coaching behaviors, teammate relationships, organizational culture, and other factors on athletes' psychological resilience and coping strategies.

## Data Availability

The original contributions presented in the study are included in the article/Supplementary material, further inquiries can be directed to the corresponding author.
