# Corrigendum: The effect of athletes' training satisfaction on competitive state anxiety—a chain-mediated effect based on psychological resilience and coping strategies

**DOI:** 10.3389/fpsyg.2025.1560457

**Published:** 2025-02-25

**Authors:** Xiaomei Yu, Yang Yang, Bo He

**Affiliations:** ^1^College of Physical Education, Chengdu Sport University, Eastern New District, Chengdu, China; ^2^College of Physical Education and Health Management, Chongqing University of Education, Nan'an District, Chongqing, China; ^3^College of Physical Education, Chongqing University of Posts and Telecommunications, Nan'an District, Chongqing, China

**Keywords:** training satisfaction, psychological resilience, coping strategies, competitive state anxiety, chain mediation

In the published article, there was an error in affiliations 1, 2, and 3. Instead of “1. School of Physical Education, Chengdu University of Physical Education, Chengdu, Sichuan, China, 2. School of Physical Education and Health Management, Chongqing Second Normal University, South Bank, Chongqing, China, 3. School of Physical Education, Chongqing University of Posts and Telecommunications, South Bank, Chongqing. China”, it should be “1. College of Physical Education, Chengdu Sport University, Eastern New District, Chengdu, China, 2. College of Physical Education and Health Management, Chongqing University of Education, Nan'an District, Chongqing, China, 3. College of Physical Education, Chongqing University of Posts and Telecommunications, Nan'an District, Chongqing, China”.

The authors apologize for this error and state that this does not change the scientific conclusions of the article in any way. The original article has been updated.

