# Supplementary material for: The effect of athletes' training satisfaction on competitive state anxiety—a chain-mediated effect based on psychological resilience and coping strategies
Source: Front Psychol. 2024 Sep 20;15:1409757. doi: 10.3389/fpsyg.2024.1409757 (PMC11450333; doi:10.3389/fpsyg.2024.1409757)
Supplement: Supplementary file 1 [file Table_1.DOCX]

Table 1 Regression equation after exchanging mediating variables

| Regression equation (N=447) | | Fitting index | | | Coefficient and significance | |
| --- | --- | --- | --- | --- | --- | --- |
| Outcome variables | Predictor variables | R | R^2^ | F | β | t |
| Coping strategies | Constants | 0.660 | 0.436 | 343.851 | 0.078 | 1.440 |
|  | Training satisfaction |  |  |  | 1.005*** | 18.543 |
| Psychological resilience | Constants | 0.865 | 0.749 | 662.140 | -0.321 | -1.349 |
|  | Training satisfaction |  |  |  | 0.312*** | 9.852 |
|  | Coping strategies |  |  |  | 0.412*** | 19.807 |
| Competitive state anxiety | Constants | 0.855 | 0.730 | 400.135 | 0.007 | 0.271 |
|  | Training satisfaction |  |  |  | -0.392*** | -10.804 |
|  | Coping strategies |  |  |  | -0.086*** | -2.907 |
|  | Psychological resilience |  |  |  | -0.419*** | -8.345 |
| ***p<0.001,**p<0.05 | | | | | | |

Table 2 Bootstrap analysis of mediation effect test after replacing mediator variables

|  | Effect | Boot SE | Boot LL CI | Boot UL CI | Relative mediation effect |
| --- | --- | --- | --- | --- | --- |
| Total Indirect effect | -0.385 | 0.024 | -0.431 | -0.336 | 49.61% |
| Indirect effect 1 | -0.087 | 0.033 | -0.152 | -0.024 | 11.21% |
| Indirect effect 2 | -0.128 | 0.022 | -0.173 | -0.087 | 16.49% |
| Indirect effect 3 | -0.170 | 0.023 | -0.216 | -0.126 | 21.91% |
| Indirect effect 1：Training satisfaction→coping strategies→competitive state anxiety  Indirect effect 2：Training satisfaction→psychological resilience→competitive state anxiety  Indirect effect 3：Training satisfaction→coping strategies→psychological resilience→competitive state anxiety | | | | | |
